# Promoting Neurovascular Recovery in Aged Mice after Ischemic Stroke - Prophylactic Effect of Omega-3 Polyunsaturated Fatty Acids

**DOI:** 10.14336/AD.2017.0520

**Published:** 2017-10-01

**Authors:** Mengfei Cai, Wenting Zhang, Zhongfang Weng, R. Anne Stetler, Xiaoyan Jiang, Yejie Shi, Yanqin Gao, Jun Chen

**Affiliations:** ^1^State Key Laboratory of Medical Neurobiology and Institute of Brain Sciences, and Collaborative Innovation Center, Fudan University, Shanghai 200032, China; ^2^Pittsburgh Institute of Brain Disorders & Recovery and Department of Neurology, University of Pittsburgh School of Medicine, Pittsburgh, PA 15213, USA; ^3^Geriatric Research, Education and Clinical Center, Veterans Affairs Pittsburgh Health Care System, Pittsburgh, PA 15261, USA

**Keywords:** docosahexaenoic acid, eicosapentaenoic acid, angiogenesis, neurogenesis, white matter restoration

## Abstract

The aged population is among the highest at risk for ischemic stroke, yet most stroke patients of advanced ages (>80 years) are excluded from access to thrombolytic treatment by tissue plasminogen activator, the only FDA approved pharmacological therapy for stroke victims. Omega-3 polyunsaturated fatty acids (n-3 PUFAs) robustly alleviate ischemic brain injury in young adult rodents, but have not yet been studied in aged animals. This study investigated whether chronic dietary supplementation of n-3 PUFAs protects aging brain against cerebral ischemia and improves long-term neurological outcomes. Aged (18-month-old) mice were administered n-3 PUFA-enriched fish oil in daily chow for 3 months before and up to 8 weeks after 45 minutes of transient middle cerebral artery occlusion (tMCAO). Sensorimotor outcomes were assessed by cylinder test and corner test up to 35 days and brain repair dynamics evaluated immunohistologically up to 56 days after tMCAO. Mice receiving dietary supplementation of n-3 PUFAs for 3 months showed significant increases in brain ratio of n-3/n-6 PUFA contents, and markedly reduced long-term sensorimotor deficits and chronic ischemic brain tissue loss after tMCAO. Mechanistically, n-3 PUFAs robustly promoted post-ischemic angiogenesis and neurogenesis, and enhanced white matter integrity after tMCAO. The Pearson linear regression analysis revealed that the enhancement of neurogenesis and white matter integrity both correlated positively with improved sensorimotor activities after tMCAO. This study demonstrates that prophylactic dietary supplementation of n-3 PUFAs effectively improves long-term stroke outcomes in aged mice, perhaps by promoting post-stroke brain repair processes such as angiogenesis, neurogenesis, and white matter restoration.

Ischemic stroke is a major cause of disability in the elderly worldwide, and with prolonged life expectancies, the aged population is significantly growing to represent a significant stroke population. Following an ischemic event, older individuals are more likely to have severe neurological disorders, larger infarct volume, higher morbidity and mortality rate [1, 2]. However, intravenous thrombolysis is restricted in aging patients due to the higher risk for intracranial hemorrhage and poorer outcomes associated with higher mortality [2, 3], and thus aging is regarded as possible exclusion criteria in patients with ischemic stroke [3-5].

Other than endovascular clot removal, which is hampered by surgical site accessibility [6], no effective therapeutic strategy is available for the treatment of aged individuals. Previous studies have demonstrated that the young adult brain possesses the ability to initiate repair processes itself following ischemic stroke [7-10]. However, ischemia-induced angiogenesis and neurogenesis is decreased in the aged rodents, characterized by less basal cell proliferation in the subventricular zone, and a lower number of stroke-generated granule cells in the dentate subgranular zone [8, 11]. In addition, ischemic stroke leads to axonal degeneration and demyelination in aged mice, which is associated with worsened neurobehavioral outcomes [12, 13]. Thus, it is necessary to investigate accessible strategies to protect against ischemic stroke in aging animals.

Omega-3 polyunsaturated fatty acids (n-3 PUFAs) are widely known for their critical role in neurodevelopment and are essential for optimal neurological function. Docosahexaenoic acid (DHA) is enriched in neuronal tissue, oligodendrocytes, and also subcellular particles such as mitochondria [14, 15]. During human aging, the content of DHA decreases in the human orbitofrontal cortex [16]; similarly, several studies in rodents have suggested that the brain is prone to lose DHA as a natural aging process [17]. Supplementation of PUFAs reverses age-related decrease of DHA absorption-related receptors, increases hippocampal neurogenesis, dendritic arborization of newborn neurons and neuronal density, and is associated with decreased cognitive decline during aging [18, 19]. Additionally, dietary consumption of fish, a major source of n-3 PUFAs, is correlated with larger volume of gray matter, and lower prevalence of subclinical infarct [20-22]. Our previous research using either dietary supplementation or transgenic overexpression of *fat-1* to bolster systemic n-3 PUFAs before ischemic injury in mice suggests that cerebral accumulation of n-3 PUFAs upregulated expression of angiopoietin 1 and angiopoietin 2, which contribute to enhanced angiogenesis, neurogenesis, and oligodendrogenesis after ischemic stroke [23-25]. However, no studies have determined the effects of increased n-3 PUFAs in aged animals. Given the context that aging itself impacts n-3 PUFAs content in the brain, and that ischemic stroke elicits more severe injury in aged animals, we hypothesized that dietary supplementation with n-3 PUFAs would greatly improve stroke outcomes in aged mice. Here we show that chronic dietary administration of n-3 PUFAs robustly increases the concentration of n-3 PUFAs in brain parenchyma in aging brain and protects brain against transient cerebral ischemia up to 56 days post insult. Furthermore, n-3 PUFAs significantly enhanced post-stroke angiogenesis, neurogenesis, and protected white matter integrity. Thus, prophylactic administration of n-3 PUFA-enriched fish oil in aged individuals may serve as a potential and promising therapeutic candidate for preventing neurobehavioral disorders induced by cerebral ischemia and stimulating restoration of neurovascular unit and white matter integrity in the aged brain after stroke.

## MATERIALS AND METHODS

### Dietary supplementation with fish oil

All experimental procedures were approved by the University of Pittsburgh Institutional Animal Care and Use Committee and Animal Care and Use Committee at Fudan University, and performed in accordance with the *National Institutes of Health Guide for the Care and Use of Laboratory Animals*. Eighteen-month-old male C57BL/6J mice were either fed a standard laboratory rodent diet (containing low n-3 PUFAs concentration (0.5%)) or the same chow supplemented with n-3 PUFAs (docosahexaenoic and eicosapentaenoic acids, triple strength n-3 fish oil, Puritan’s Pride, Oakdale, NY, USA, final n-3 PUFAs concentration 4%) for 3 months before exposure to transient cerebral ischemia and up to 56 days after ischemia until sacrifice. All animals were randomly assigned to each experimental group. Stroke model surgery and all outcome analyses were performed in a blinded manner.

### Lipid extraction and fatty acid analysis

Brain fatty acid contents were measured in two groups of randomly assigned mice either under the standard laboratory rodent diet (N3L) or with supplementation of n-3 PUFAs (N3H) for 3 months without stroke induction. The cerebral cortex from extracted brains were dissected, snap frozen and dried into powder in a vacuum freeze dryer. Fatty acids were extracted as described previously [26]. Fatty acid composition was determined by capillary gas chromatography using a Clarus 500 Gas Chomatograph (Perkin Elmer, Waltham, Massachusetts, USA). Tissue fatty acid methyl ester peak identification was performed by comparison to the peak retention times of a 30-component methyl ester standard (Sigma-Aldrich, St. Louis, Missouri, USA). The concentration of each fatty acid was determined by calculating peak areas. Four animals were analyzed in each group.

### Transient focal cerebral ischemia model and infarct volume measurements

Transient focal cerebral ischemia was induced in aged male mice (21 months old, 30-35g) by intraluminal occlusion of the left middle cerebral artery (MCAO) for 45 minutes, and experimental procedures were performed following Stroke Therapy Academic Industry Roundtable (STAIR) guidelines [27]. Animals were initially anesthetized with 3% isoflurane vaporized in 30% O_2_/70% N_2_ until they were unresponsive to the tail pinch test, and then maintained under anesthesia with 1.5% isoflurane. A monofilament (8-0) with a silicone-coated tip was introduced into the common carotid artery, advanced to the origin of the MCA, and left in place for 45 minutes. Rectal temperature was maintained at 37±0.5°C during surgery with a temperature-controlled heating pad. Regional cerebral blood flow (rCBF) was measured using laser doppler; animals that did not show an rCBF reduction of at least 75% of the baseline levels or that died after ischemia induction were excluded from the study. Sham-operated mice underwent the same anesthesia and surgical procedures but without MCAO.

### Assessment of neurological functions

Animals underwent pretraining for three days before surgery to establish a baseline neurological function, and then were subjected to testing 3-35 days after ischemia. All tests were performed by investigators blinded to experimental groups. Sensorimotor deficits were assessed by the corner test and cylinder test. The corner test was used to determine the number of left turns out of 10 turn trials per day as a measure of functional symmetry. The cylinder test was performed to assess symmetry in forepaw use. Mice were individually placed in a transparent cylinder (height: 15 cm, diameter: 9 cm) and videotaped for 15 minutes. Initial forepaw use in making contact against the cylinder wall after rearing was then recorded. Asymmetry was expressed as the relative proportion of right forepaw contacts, and calculated as follows: (left - right) / (left + right + both).

Animals that survived all neurobehavioral tests were further kept in the protocol up to 56 days after MCAO for various histological and immunohistochemical assessments described below. Total 11 mice survived the 56-day long-term study (N3L group, n=5 mice; N3H group, n=6 mice).

### BrdU labeling of proliferating cells

The S-phase marker 5-bromo-2-deoxyuridine (BrdU) was used to label cells that underwent proliferation at the time of BrdU injection. BrdU (50 mg/kg body weight, Sigma-Aldrich) was injected intraperitoneally daily beginning 3 days after MCAO and continuing until 10 days after MCAO. Animals were subsequently deeply anesthetized and transcardially perfused with 0.9% NaCl followed by 4% paraformaldehyde in phosphate buffer. Brains were then sequentially cryoprotected in 20% and 30% sucrose, and frozen serial coronal brain sections (25 μm) were prepared on a cryostat (Leica, Bensheim, Germany). Sections were pretreated with 2N HCl for 1 hour at 37°C, followed by 0.1M boric acid (pH 8.5) for 10 minutes at room temperature, and then blocked with the M.O.M kit (Vector, Brulingame, CA, USA), followed by incubation with mouse anti-BrdU antibody (1:1000, BD bioscience, San Jose, CA, USA) for 1 hour at room temperature and overnight at 4°C. After a series of washes, sections were then incubated in 594 or 488-conjugated goat anti-mouse IgGs (1:1000, Jackson ImmunoResearch Laboratories, Inc. West Grove, PA, USA) for 1 hour at room temperature, rinsed, counterstained with DAPI for visualization of nuclei, and coverslipped for microscopic evaluation.

### Vascular labeling and analysis of vascular density

Vessels were visualized by CD31 staining at 56 days after MCAO. Free floating sections were prepared from fixed and dehydrated brains and stained with CD31 antibody (1:500, Santa Cruz Biotechnology). After incubation in primary antibody for 1 hour at room temperature and overnight at 4°C, coronal brain sections were incubated with 488-conjugated goat anti-rabbit (1:1000, Jackson ImmunoResearch Labortories, Inc) secondary antibodies to stain vessels. Four sections at 0.1 mm intervals were analyzed in each brain. The number of CD31^+^ vessels longer than 50 μm was counted in the ipsilateral peri-infarct striatum. The vascular length per mm^2^ was calculated using ImageJ software by a blinded observer.

### Measurement of tissue loss

Tissue loss was assessed by microtubule-associated protein 2 (MAP2) staining at 56 days after MCAO. Free floating sections were prepared from fixed and dehydrated brains and stained with MAP2 antibody (1:500, Abcam). Infarct volume was determined with NIH ImageJ analysis by an observer blinded to the experimental group assignment. The noninfarcted area in ipsilateral hemisphere was measured by substracting MAP2-stained area of the ipsilateral side from that of contralateral side. The percentage of tissue loss was determined using the following equation: (volume of the contralateral hemisphere ? noninfarcted volume of the ipsilateral hemisphere)/ (volume of the contralateral hemisphere) × 100%.

### Immunohistochemical staining

Immunohistochemical staining was performed on 25 μm-thick free-floating sections. Briefly, coronal brain sections were blocked with 5% goat serum in phosphate-buffered saline (PBS) with 0.1% Triton-X 100 for 1 hour, followed by primary antibody incubation for 1 hour at room temperature and overnight incubation at 4°C. Mature neurons were visualized using rabbit monoclonal anti-NeuN (1:1000; Abcam) antibody. After a series of washes, sections were incubated for 1 hour at room temperature with goat-anti-rabbit secondary antibodies conjugated with DyLight 594 (1:1000, Jackson ImmunoResearch Labortories, Inc). Sections were mounted and coverslipped with Fluoromount-G (Southern Biotech, Birmingham, AL, USA). Immature neural progenitor cells were visualized using anti-doublecortin (DCX, 1:500; Santa Cruz Biotechnology, Santa Cruz, CA) followed by Cy3-conjugated secondary IgG antibody (1:1000, Jackson Immunoresearch Laboratories). Myelin and abnormally dephosphorylated neurofilaments were visualized with anti-myelin basic protein (MBP, 1:500; Abcam) and anti-SMI-32 (1:1000, Millipore, Billerica, MA) primary antibodies, respectively. BrdU/NeuN and BrdU/DCX double stained cells were calibrated in three randomly selected regions of interest (ROIs) within peri-infarct area of striatum. Angiogenesis was evaluated by BrdU immunopositive cells along the CD31 labeled microvessels [28]. To assess the ratio of SMI-32/MBP, two microscopic fields from striatum and one microscopic field from corpus callosum in peri-infarct region were selected from each ischemic brain. The regions of interested in the sham brain and contralateral hemisphere were the same as that in the ipsilateral side of N3L-fed mice. Fluorescence intensity was measured using NIH ImageJ analysis by an observer blinded to the experimental group assignment. SMI-32/MBP ratio in the ipsilateral hemisphere was compared with contralateral hemisphere. Fluorescence images were captured by a blinded observer.

**Figure 1.**
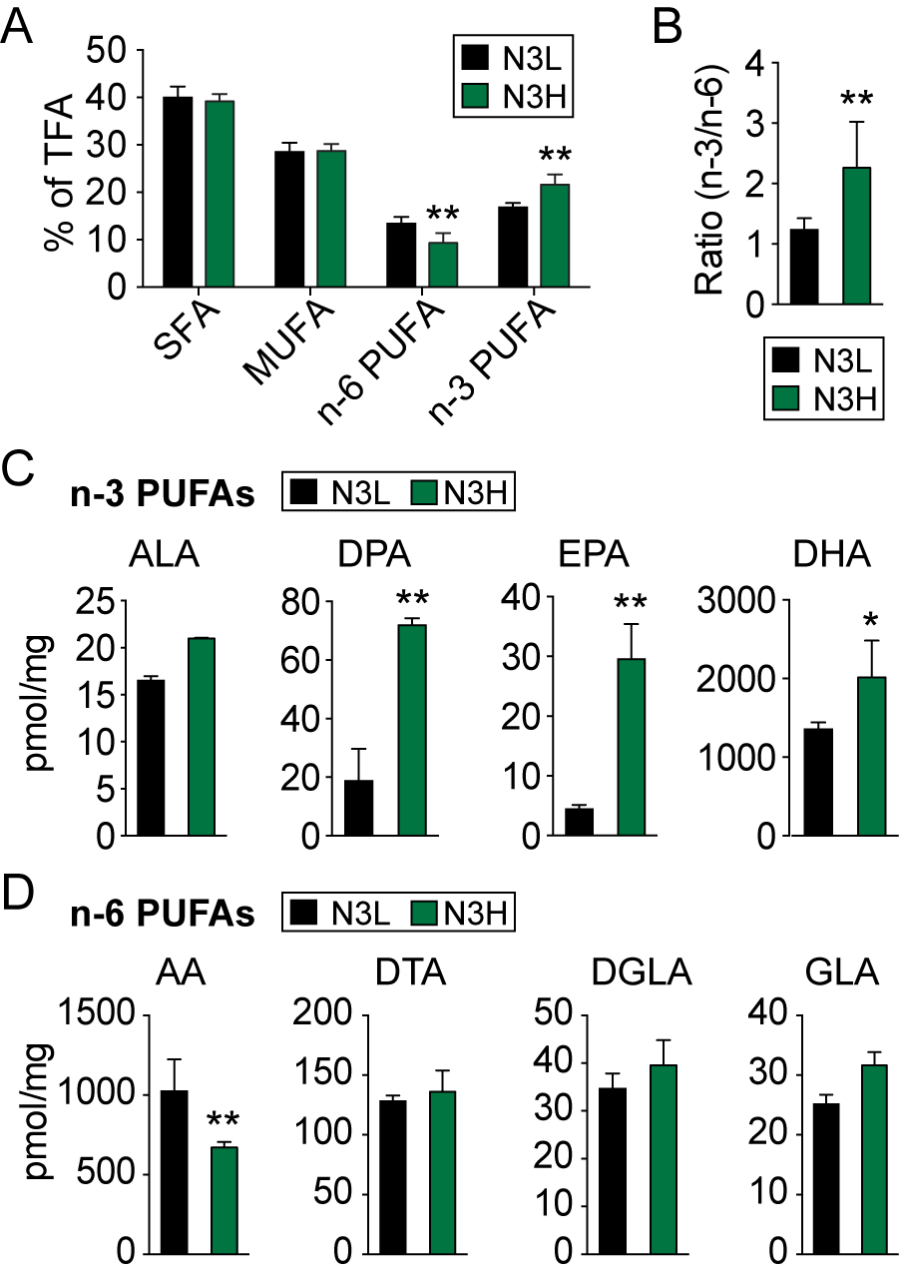
Lipid profiles are altered in the forebrains of aged mice by dietary PUFA supplementation Mice were maintained on a low n-3 PUFA (N3L) or high n-3 PUFA (N3H) diet for 3 months, and forebrains were then processed for lipid analysis. **(A)** Lipid profiles in mouse forebrains expressed as the percent of total fatty acids (TFA), and included profiles of saturated fatty acids (SFA), mono-unsaturated fatty acids (MUFA), and poly-unsaturated fatty acids (PUFA). **(B)** The ratio of forebrain n-3 to n-6 fatty acids increased in N3H-fed mice. **(C)** Specific n-3 PUFAs content expressed as pmol/mg; the n-3 PUFAs include α-linolenic acid (ALA), docosapentaenoic acid (DPA), eicosapentaenoic acid (EPA), and docosahexaenoic acid (DHA). **(D)** Specific n-6 PUFAs content expressed as pmol/mg: the n-6 PUFAs include arachidonic acid (AA), docosatetraenoic acid (DTA), dihomo-γ-linolenic acid (DGLA) and γ-linoleic acid (GLA). Data are mean ± SEM, n=4 per group; *p≤0.05, **p≤0.01 vs. N3L.

### Statistical analysis

All data are reported as the mean ± SEM. Significant differences between means were assessed by ANOVA and *post hoc* Scheffe tests for multiple comparisons. The Pearson product linear regression analysis was used to correlate the indicating histological parameters with the sensorimotor functions in aged mice. A *p* value of less than 0.05 was deemed statistically significant.

**Figure 2.**
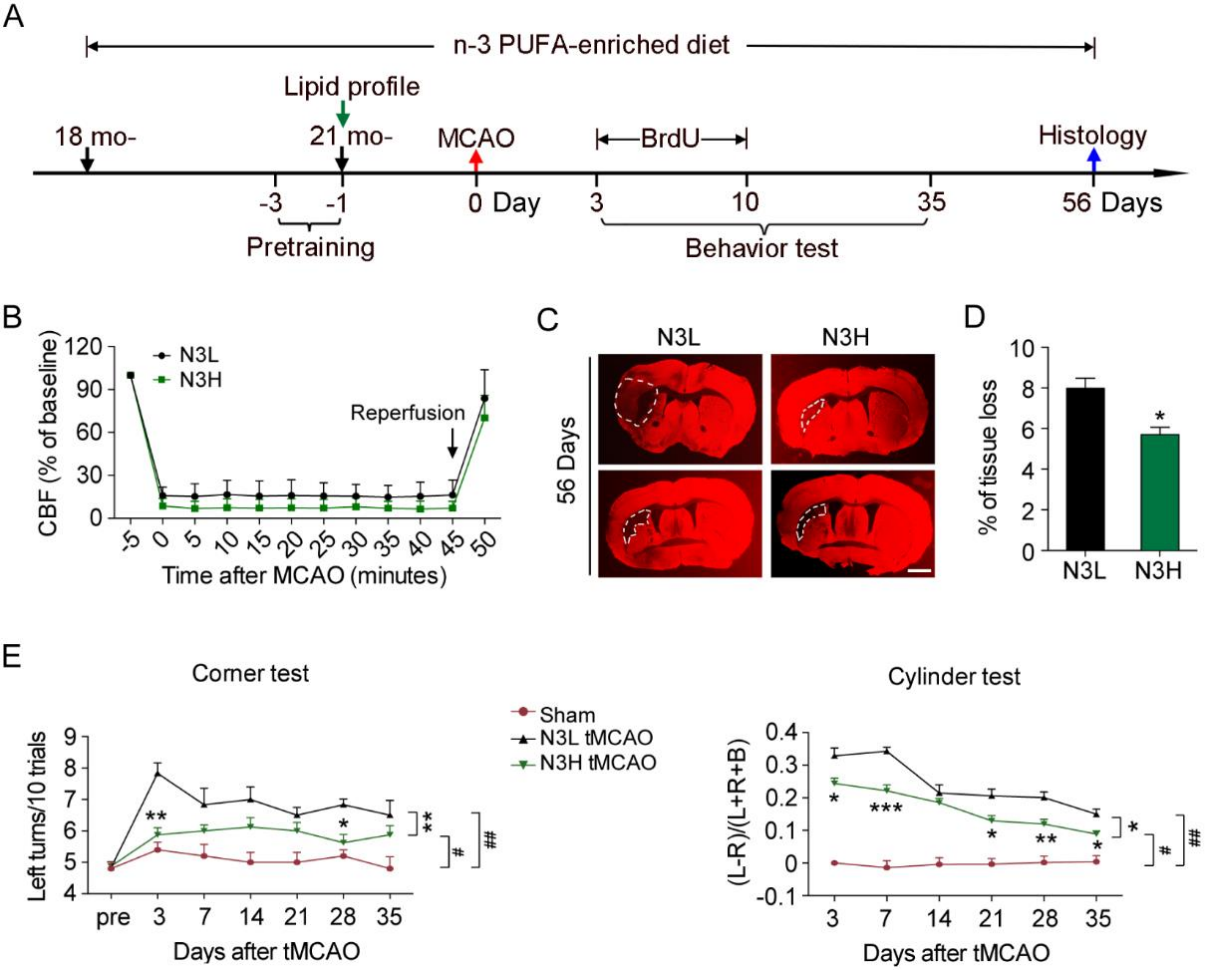
Dietary n-3 PUFAs supplementation protects against long-term behavioral deficits and infarct induced by ischemic brain injury in aged mice **(A)** Diagram of the experimental timeline. 18-month-old (18 mo) mice were fed either the standard chow or chow supplemented with n-3 PUFAs enriched fish oil, then subjected to 45 min of transient MCAO at 21 months (21 mo) of age. After MCAO, the mice were maintained on the same diet as prior to MCAO until the end of the study (56 days after MCAO). Pre-training for behavioral tests occurred 3 days before 45 min transient MCAO or sham operation. BrdU was injected daily starting 3 days after MCAO through day 10 after MCAO. Sensorimotor function was evaluated up to 35 days after MCAO. Mice were sacrificed 56 days after MCAO for histological assessments. **(B)** Regional cerebral blood flow measured by laser Doppler during and after MCAO showed no difference between N3L and N3H groups for the duration and extent of ischemic induction. (**C, D**) Tissue loss from N3L- and N3H-fed mice 56 days after MCAO was measured by MAP2 immunostaining. Representative images of MAP2 staining at 56 days after MCAO are shown, where the dashed lines illustrate chronic brain infarct (MAP2-negative area). Scale bar=1mm. **(E)** The corner test and cylinder test performance over 35 days after MCAO demonstrated impairments in both groups of MCAO mice. The N3H-fed mice showed significantly improved performance compared to ischemic mice fed the N3L diet. All data are presented as mean ± SEM, n=7 per group, #*p*≤0.05, ##*p*≤0.01 vs.; *p≤0.05, **p≤0.01 vs. N3L tMCAO.

## RESULTS

### Chronic administration of fish oil increases n-3 PUFAs in the brain of aged mice

Dietary supplementation with administration of fish oil into the standard chow has previously been demonstrated to alter the ratio of n-3 PUFAs to n-6 PUFAs in brains of young adult mice [23]. To assess whether the n-3 to n-6 PUFAs ratio can be shifted by dietary supplementation in aged mice, fatty acids were analyzed in the forebrains of n-3 PUFA-supplemented (“N3 high” or N3H) and control diet-fed (“N3 low” or N3L) mice using gas chromatography beginning three months after the initiation of the diet regimen. The proportion of the saturated fatty acids and mono-unsaturated fatty acids did not differ between N3H- and N3L-fed mice (Fig. 1A). However, the n-3 PUFAs fraction from the forebrain of N3H-fed mice was significantly increased compared to N3L-fed mice, and the n-6 PUFAs fraction was decreased in N3H-fed mice (Fig. 1A), leading to an overall increase in the n-3/n-6 ratio in the forebrain of N3H aged mice (Fig. 1B). The three major n-3 PUFAs (EPA (eicosapentaenoic acid, C20:5), DPA (docosapentaenoic acid, C22:5) and DHA (docosahexaenoic acid, C22:6)) were all significantly increased in the forebrain of N3H mice compared to N3L mice (Fig. 1C). The major n-6 fatty acids, AA (arachidonic acid, C20:4) were decreased in N3H mice compared to N3L mice (Fig. 1D). These results demonstrate that dietary intake of n-3 PUFAs is an effective means of increasing the n-3/n-6 ratio in the brain of 21-month-old mice.

**Figure 3.**
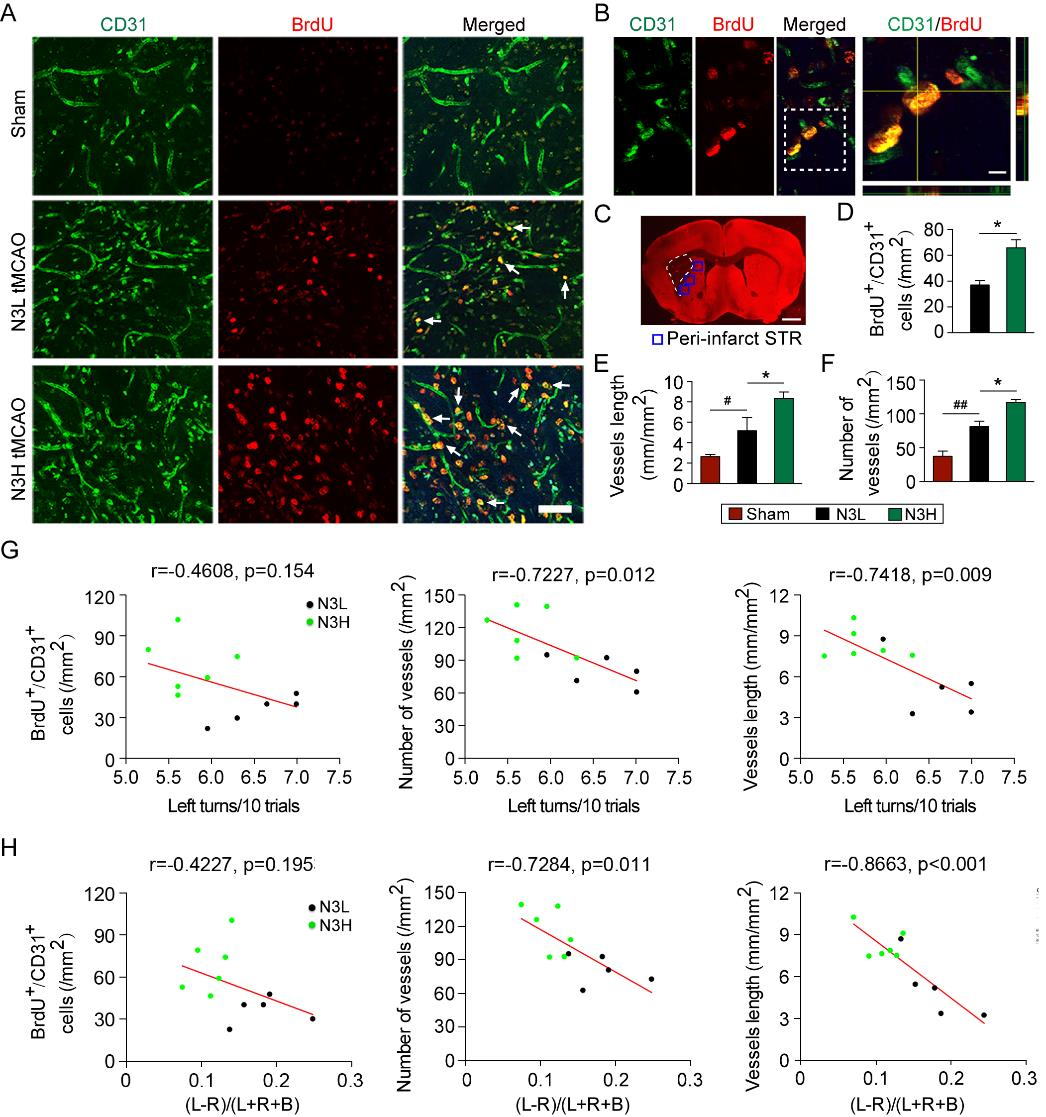
Dietary n-3 PUFAs enhances angiogenesis after MCAO in aged mice Mice were injected with BrdU daily over 3-10 days following 45 min MCAO, then processed for immunohistology for BrdU and CD31 at 56 days following MCAO. **(A)** Representative images of CD31 and BrdU double-labeling within the peri-infart region (striatum) of vehicle and n-3 PUFAs treated brains at 56 days after MCAO. *Arrow*: BrdU^+^/CD31^+^ cells. Scale bar=50 μm. **(B)** Representative high power confocal images of BrdU^+^/CD31^+^ co-localization in the striatum at 56 days after MCAO. Scale bar=10 μm. **(C)** An image stained with MAP2 to designate the infarct area. Analysis of angiogenesis was derived from the regions marked by the blue boxes in the peri-infarct striatum. **(D-F)** Quantification of post-stroke generated (BrdU^+^/CD31^+^) vessels, vessel (CD31^+^) length, and number of all vessels (CD31^+^ vessels) at 56 days after cerebral ischemia. Data are presented as mean ± SEM, N3L group, n = 5; N3H group, n=6. *p≤0.05 vs. N3L, #*p*≤0.05, ##*p*≤0.01 vs. sham. **(G, H)** Pearson linear regression analysis was performed to correlate the performance of corner test (G) and cylinder test (H) at 21-35 days after MCAO with the number of BrdU^+^/CD31^+^ cells in striatum at 56 days after MCAO.

### n-3 PUFAs reduce tissue loss and confer long-term neuroprotection against cerebral ischemia

Aged animals have worsened outcomes following ischemic injury, including increased mortality [12, 29]. To determine the effects of n-3 PUFA dietary supplementation on ischemic stroke in aged animals, 21-month-old mice fed either the N3H or the N3L diet were subjected to a less severe transient ischemic model (45 min of MCAO) and assessed for behavior and histological outcomes up to 56 days after ischemia (Fig. 2A). The duration and degree of ischemia did not differ between treatment groups as measured by laser Doppler in the ipsilateral hemisphere (Fig. 2B). In addition, no discernible differences were found among sham-operated animals, regardless of the diet on which they were maintained (data not shown). Due to this observation, sham animals were pooled. Since this study intended to correlate various histological assessments with sensorimotor outcomes after stroke, only animals that underwent all neurobehavioral tests and survived 56 days after MCAO (5 mice in the N3L group, 6 mice in the N3H group) were included in statistical analyses. MAP2 immunostaining was performed for analysis of tissue loss. N3H-fed mice exhibited significantly smaller tissue loss than N3L-fed mice 56 days following cerebral ischemia (Fig. 2C, D). Sensorimotor outcomes were assessed over 35 days after the onset of stroke using cylinder and corner tests (Fig. 2E). Animals with sham operations displayed similar neurobehavioral performance regardless of diet that did not change significantly over time. After cerebral ischemia, N3L- and N3H-fed mice both showed spontaneous recovery in sensorimotor function within 35 days of reperfusion. However, neurological deficits induced by cerebral ischemia were significantly less pronounced in N3H mice compared to N3L ones, according to these two behavioral tests. Thus, amelioration of n-3 PUFAs in the brain was correlated with improved survival and long-term sensorimotor function after ischemic insult.

**Figure 4.**
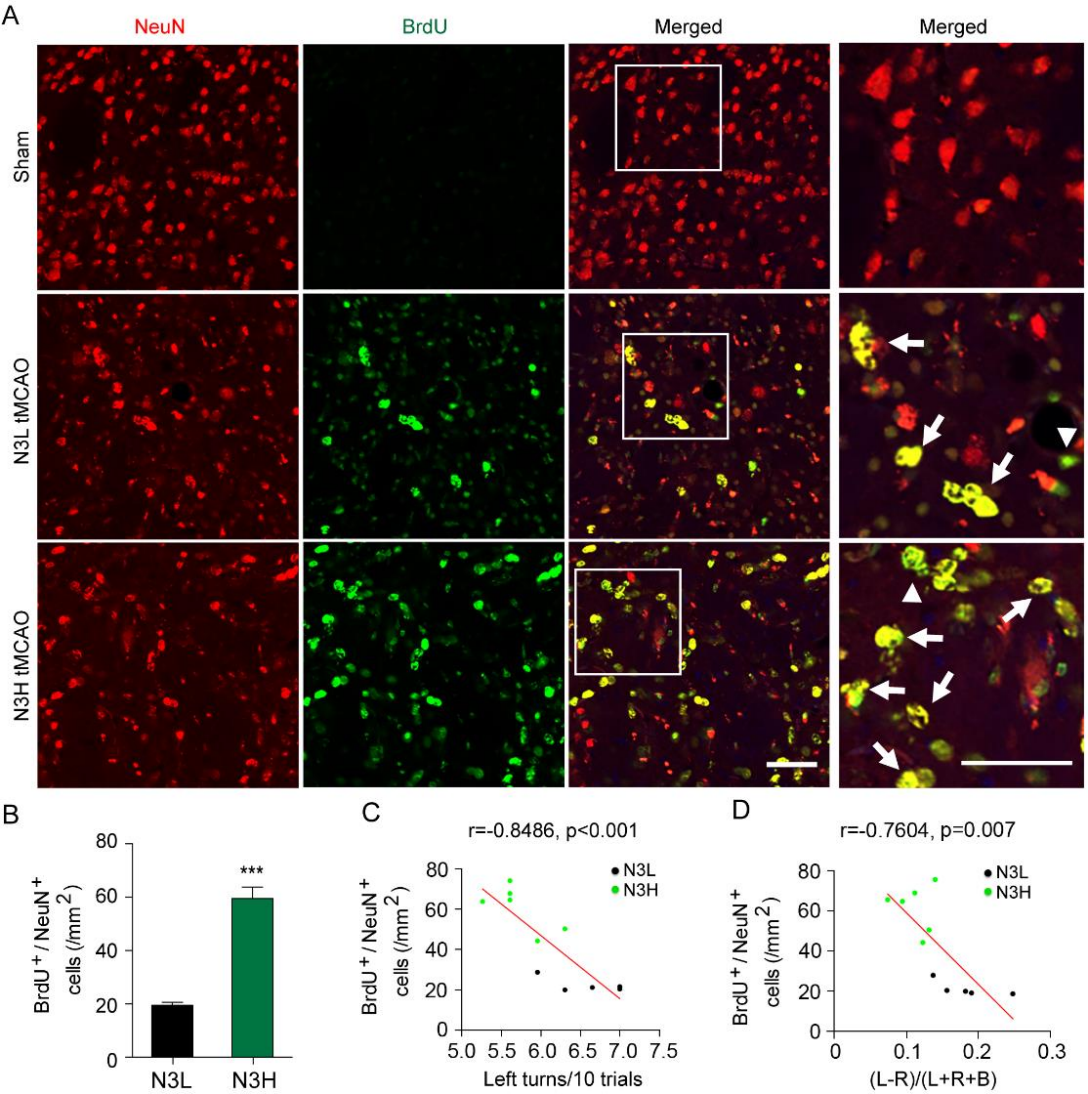
Dietary supplementation of n-3 PUFAs increase the presence of matured neural progenitor cells after ischemia in aged mice **(A)** Representative images of mature neurons (NeuN^+^, red) and BrdU^+^ (green) cells in the striatum after MCAO. Scale bar=50 μm. *Arrow*: NeuN^+^/BrdU^+^ cells. **(B)** Quantification of BrdU^+^/NeuN^+^ cells at 56 days after cerebral ischemia. Data are presented as mean ± SEM, n = 5-6 mice per group at each time point. ***p≤0.001 vs. N3L. **(C, D)** Pearson linear regression analysis was performed to correlate the performance of corner test **(C)** and cylinder test **(D)** at 21-35 days after MCAO with the number of NeuN^+^/BrdU^+^ cells in striatum at 56 days after MCAO. N3L group, n = 5; N3H group, n=6.

**Figure 5.**
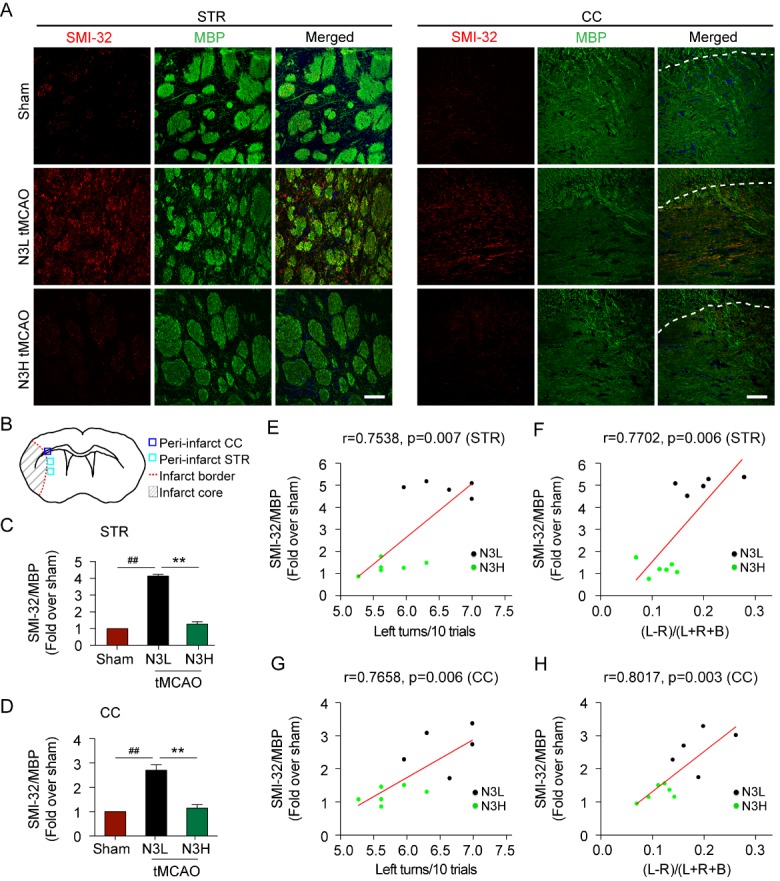
n-3 PUFAs supplementation decreases demyelination after MCAO in aged mice **(A)** Representative images of SMI-32 and MBP double staining in striatum (STR) and in corpus callosum (CC) of aged mice 56 days after MCAO. The dashed white line indicates the border between the cortex and corpus callosum. Scale bar=50 μm. **(B)** Diagram to indicate the infarct core, infarct border and peri-infarct regions. The blue and purple boxes indicate the areas used for histological assessments for STR and CC, respectively. **(C, D)** Quantification of SMI-32/MBP ratio in the striatum **(C)** and corpus callosum **(D)**. Data are presented as mean ± SEM, n = 5 per group, **p≤0.01 vs. N3L. ##*p*≤0.01 vs. sham. **(E-H)** Pearson linear regression analysis was performed to correlate asymmetric rate of forelimb use in corner test **(E, G)** and cylinder test **(F, H)** at 21-35 days after MCAO with the ratio of SMI-32/MBP in STR **(E, F)** or CC **(G, H)** 56 days after MCAO. N3L group, n = 5; N3H group, n=6.

### n-3 PUFAs dietary supplementation enhances angiogenesis and neurogenesis in the ischemic brain

The aged brain is thought to have diminished capacity for remodeling *via* cellular proliferation and differentiation for the repair of both the vasculature and neuronal networks after injury [8, 12]. Our previous studies suggest that, in the young ischemic brain, n-3 PUFAs can stimulate angiogenesis and neovascularization after cerebral ischemia in young adult mice, and that this stimulation of blood flow can improve neurogenesis and migration [23]. However, whether n-3 PUFAs supplementation can overcome the limitations of aging and promote revascularization, angiogenesis, and neurogenesis after ischemia in aged mice is currently unknown. To address this issue, we injected BrdU daily to identify newly formed cells generated 3-10 days following ischemia, and then co-immunostained for BrdU and CD31 to identify mature vessels present at 56 days after stroke (Fig. 3A, B). Vessels were histologically analyzed from the peri-infarct region in the striatum (blue boxes, Fig. 3C). Ischemia alone (N3L-fed ischemic mice) induced a significant increase in the number of newly generated vessels (BrdU^+^/CD31^+^) cells, total vessel length and total vessel number compared to sham (Fig. 3D–F), indicating that an innate angiogenic response occurs in the aged brain that persists to 56 days following ischemic injury. Supplementation with dietary n-3 PUFAs further potentiated the number of BrdU-positive vessels, vessel length, and total number of vessels in the peri-infarct striatum of N3H-fed mice compare to N3L-fed mice at 56 days after cerebral ischemia (Fig. 3D–F). No significant difference was detected between N3L and N3H animals with sham operations (data not shown). Although the number of BrdU^+^/CD31^+^ cells did not appear to correlate with sensorimotor performance, the increase in the total number of vessels and longer vessel length both demonstrated a positive correlation with improved sensorimotor outcomes (Fig. 3G, H). Thus, dietary supplementation with n-3 PUFAs can elicit a robust angiogenic response above the endogenous remodeling that occurs following ischemic injury in aged mice, and is positively correlated with improved sensorimotor outcomes.

In young mice, ischemic injury stimulates remodeling of the injured brain not just for the purpose of reperfusion, but also as an effort to restore neuronal networks [30]. During neurogenesis, neural stem cells proliferate in the subventricular zone (SVZ), and of these, some neuroblasts migrate towards the ischemic area to differentiate into mature neurons and incorporate into pre-formed neuronal network [31, 32]. Our previous studies suggest that an intact vascular network is critical for supporting the migration of neuroblasts, as most of the migrating neuroblasts move along blood vessels towards the ischemic area in adult mice [23]. To determine the effect of n-3 PUFAs dietary supplementation on neurogenesis and differentiation in aged mice, we immunostained ischemic tissue from aged mice injected with BrdU 3-10 days following MCAO with DCX, a marker for immature neural progenitor cells (Supplemental Fig. 1A, B). Compared with N3L mice, the number of total DCX^+^ neural progenitor cells was significantly higher in striatum of n-3 PUFAs supplemented mice 56 days following MCAO (Supplemental Fig. 1), and the DCX^+^ cells appeared to be associated with CD31^+^ vessels (Supplemental Fig. 1C). Despite the presence of more DCX^+^ cells in N3H-fed aged mice, dietary supplementation did not lead to any significant differences in the number of post-stroke (BrdU^+^) DCX cells between N3L and N3H mice at 56 days after cerebral ischemia (Supplemental Fig. 1D). In addition, no significant correlation existed between the number of DCX^+^ cells and behavioral outcomes (corner test r=-0.5124, p=0.107 and cylinder test r=-0.5063, p=0.112, Supplemental Fig. 1E, F).

Given that DCX is a marker of immature neuroblasts, we next determined whether n-3 PUFAs could stimulate the maturation of neural progenitor cells, which may be a stronger marker for neuronal remodeling after stroke. The neuronal marker NeuN was used in conjunction with BrdU (as described above) to visualize neurons generated after MCAO in aged mice fed either the N3L or N3H diet (Fig. 4A), and analyzed using the peri-infarct regions in the striatum (designated by the blue boxes in Fig. 3C). n-3 PUFAs enhanced the post-MCAO generation of NeuN^+^ cells up to 56 days after MCAO (Fig. 4B). Whether these neurons functionally integrated into the existing neural circuits remains unknown. Pearson linear regression analysis was performed to investigate the correlation between sensorimotor deficits with the number of surviving mature neurons (NeuN^+^) that were generated after MCAO (BrdU^+^). The number of BrdU^+^/NeuN^+^ cells displayed a significant positive correlation with better sensorimotor function assessed by corner test (Fig. 4C, r=-0.8486, p=0.0001) and cylinder test (Fig. 4D, r=-0.7604, p=0.007). These results suggest that n-3 PUFAs dietary supplementation in aged mice robustly stimulates generation, migration and maturation of neural progenitor cells, which is positively correlated with sensorimotor recovery.

### n-3 PUFAs alleviate cerebral ischemia induced white matter injury

Stroke induces severe white matter injury, which is detrimental to the electrophysiological communication between brain regions [33-35]. With increased age, white matter becomes more sensitive to ischemic injury [36, 37]. We have reported that increased levels of n-3 PUFAs prior to ischemic injury enhances the post-ischemic proliferation of immature oligodendrogenic precursor cells (OPCs), preserves the number of total OPCs, and improves white matter integrity up to 35 days after stroke in young adult animals [23]. In order to determine the effect of n-3 PUFAs supplementation on white matter recovery in aged mice, MBP (a marker of mature oligodendrotytes) and SMI-32 (a marker of nonphosphorylated neurofilament associated with axonal damage [38, 39]) were co-immunolabeled using brain sections harvested at 56 days after cerebral ischemia (Fig. 5A). White matter was assessed histologically from the peri-infarct regions of the striatal fiber bundle and the corpus callosum (designated by the boxes in Fig. 5B). The ratio of SMI-32/MBP in the ipsilateral hemisphere was compared with that of the sham. Axons in the striatum and corpus callosum are predominantly myelinated under normal conditions (Fig. 5A–D). Ischemic stroke induced oligodendrocytic damage and progressive demyelination of axons, characterized with increased SMI-32 staining and decreased MBP staining in the peri-infarct regions of the ipsilateral striatum and corpus callosum, and thus reflected by the persistent increase in the SMI-32/MBP ratio in N3L-fed aged mice at 56 days after MCAO compared to sham (Fig. 5C,D). White matter injury in aged mice fed the N3H diet was significantly less at 56 days after MCAO compared to aged mice fed the N3L diet. When tested for correlation with sensorimotor function using Pearson linear regression analyses, the ratio of SMI-32/MBP in either the peri-infarct striatum or corpus callosum was tightly associated with sensorimotor outcomes, such that increased ratio of SMI-32/MBP correlated with a greater degree of paw preference in asymmetry of corner turns (Fig. 5E, G) or the cylinder exploration (Fig. 5F, H). These data suggested that improved white matter integrity by n-3 PUFAs dietary supplementation significantly contributes to the improvement of neurological outcomes following ischemic brain injury.

## DISCUSSION

The present study is the first to investigate the therapeutic efficacy of n-3 PUFAs in aged brain following transient cerebral ischemia. Extended dietary supplementation with n-3 PUFA-enriched fish oil significantly increased the content in brain parenchyma of aging mice. n-3 PUFAs significantly improved neurological outcomes, which were positively correlated with enhanced neurogenesis, angiogenesis and white matter integrity following transient cerebral ischemia.

The aged brain contains less PUFAs content and a greater susceptibility to injury from an ischemic episode [40-43]. However, the role for PUFAs in improving stroke outcomes in aged brain, or even if the aged brain could incorporate stores of PUFAs from dietary supplementation as a prophylactic therapy against stroke injury, had remained unknown. Due to the deficiency of enzymes critical to their synthesis, PUFAs cannot be synthesized *de novo* in vertebrate tissue. In young animals, supplementation of n-3 PUFAs significantly increases their concentration in parenchyma of brain [23, 44, 45]. Supplementing 18-month-old mice with triple strength n-3 PUFAs fish oil for 3 months significantly elevated the brain content of n-3 PUFAs in aged mice.

The risk of stroke increases with the increase of age, and older patients suffer more severe functional disability and slower recovery after stroke when compared to younger patients [46, 47]. Previous studies suggest that aging dampens brain remodeling activity and results in worsened long-term neurological deficits following ischemic stroke [8, 11, 48]. Our previous study suggests that in young mice, n-3 PUFAs prompt neurogenesis, angiogenesis and oligodendrogenesis to improve neurological function after transient cerebral ischemia [23]. In the present study, we have confirmed that n-3 PUFAs supplementation in 18-month-old mice enhances an endogenous remodeling process following ischemic stroke to promote functional recovery. On this basis, dietary n-3 PUFA supplementation may be an effective therapeutic regimen for the treatment of elderly patients at risk for cerebral ischemic stroke.

Angiogenesis, characterized by newly generated endothelial cells and microvessels, is a process for the reconstruction of arteries and functional recovery following cerebral ischemia [30, 49-51]. In animal models of stroke, ischemic injury stimulates angiogenesis in the peri-infarct regions for at least two weeks in young adult brain [30, 49, 52]. In humans, the angiogenesis process occurs at 3-4 days after ischemic stroke, and a higher intensity of blood vessels in ischemic hemisphere correlates with longer survival and better neurological recovery, indicating the critical role of revascularization after ischemic insults [53]. However, a recent study has suggested that aging impedes angiogenic activity during the self-recovery process [54]. In our study, we found that the aged brain still possesses an angiogenic response after cerebral ischemia, exhibiting both increased total number and length of vessels in the peri-infarct region 56 days following MCAO (Fig. 3). Nevertheless, this response was not maximal in aged mice fed with N3L, as supplementation with n-3 PUFAs could further enhance ischemia-induced angiogenesis, and the intensity and length of cerebral blood vessels were positively correlated with improved long-term sensorimotor activities. These results support the notion that increased formation of new microvessels may contribute to functional recovery after stroke [53]. Although n-3 PUFAs robustly enhanced the proliferation of endothelial cells characterized with double staining of BrdU+ and CD31+, the number of BrdU+/CD31+ cells were not significantly correlated with sensorimotor functions (Fig. 3). Thus, the augmented proliferation of endothelial cells alone does not recapitulate the whole beneficial effect of neovascularization afforded by n-3 PUFAs. Indeed, a complete angiogenesis process involves multiple steps, including degeneration of extracellular matrix, endothelial migration and proliferation, recruitment of pericytes, and sprouting of the vessels, which eventually generates new microvessels from existing vasculature [55, 56]. Our results emphasize the importance of complete revascularization, but not endothelial cell proliferation alone, in stroke recovery.

DCX^+^ neural progenitor cells move along the perfused vessels, indicating that enhanced angiogenesis may facilitate the neurogenesis and reconstruction of neuronal network, as well as the recovery of sensorimotor functions after ischemia in aged brain [57, 58].

Neurogenesis occurs in adult mammalian brain throughout life [59, 60] and is enhanced in the young adult brain after cerebral ischemia [61-63]. However, aging has been associated with the decreased proliferation of progenitor cells and suppressed production of newborn neurons [63-65]. We found evidence for a modest increase in newly generated neurons following ischemic injury in aged mice, but the response appeared to be muted. However, neurogenesis activity was much higher in N3H-fed ischemic mice, characterized by increased number of mature neurons in ischemic penumbra area, compared to N3L ischemic aged groups. Although the level of total DCX^+^ cells increased in the peri-infarct striatum of N3H-fed mice at 56 days following ischemia, no significant difference of BrdU^+^/DCX^+^ cell numbers was detected between N3L- and N3H-fed groups, nor were DCX^+^ cell counts correlated with sensorimotor performance. The discord between the lack of correlation of n-3 PUFAs supplementation and DCX^+^ cells and the positive correlation of NeuN^+^ cell counts and functional outcomes may hinge on several factors. First, the process of neuronal remodeling involving neurogenesis may be a transient phenomenon, for which our extended time point (56 days) may be farther along in the recovery process to no longer necessitate immature neurons such as DCX^+^ cells. Secondly, it is possible that not all DCX^+^ cells differentiate into mature neurons and integrate into the neural circuits. Besides enhancing maturation of progenitor cells, later stages of remodeling may shift to support the long-term survival of newly matured neurons for the functional recovery. Our results suggest that n-3 PUFAs not only prompt maturation of neural progenitor cells, but that the presence of these newly generated mature neurons improves long-term sensorimotor performance after ischemia in aged mice.

The molecular mechanisms of n-3 PUFAs on neurogenesis remain to be illustrated. DHA, the major n-3 PUFAs present in brain, can influence neuron functions through multiple mechanisms, including membrane fluidity, flexibility, signal transduction, gene expression and formation of lipid rafts [66-69]. A previous report suggests that the neuronal precursor population in the hippocampus remains stable into old age, but the increased levels of corticosteroids suppress neurogenesis, which may contribute to age-related memory disorders [70]. DHA significantly decreases circulating corticosteroids level [71, 72], and neuron proliferation can be restored by reducing corticosteroid levels in aged rodents [70]. To extrapolate from these observations, n-3 PUFAs may promote neurogenesis in aging brain after stroke via downregulating corticosteroid levels. Studies using *fat-1* transgenic mice demonstrated that DHA increases expression of several dendritic spine-related genes, which may contribute to the neurogenesis and neuritogenesis in the hippocampus [68].

Critical to preserving axonal health, oligodendrocytes in white matter can be newly generated from OPCs. Cerebral ischemia induces white matter injury, and subsequently the young adult brain mounts and endogenous effort to stimulate proliferation of OPCs and migration towards demyelinated areas in order to differentiate into mature oligodendrocytes and restore myelin sheaths. Others have suggested that the capacity for the repair of white matter injury diminishes with increased age [48, 73, 74]. Our previous studies demonstrate that n-3 PUFAs preserve the integrity of white matter and enhance oligodendrogenesis in young adult mice following ischemic insults [23, 75]. The current study uses mice that are advanced in age (21-months-old), and is the first to report a long lasting protective effect of n-3 PUFAs on white matter injury induced by ischemic stroke in aged mice. Although we did not examine the impact of n-3 PUFAs on oligodendrogenesis in aged mice, better neurological functions attribute to the integrity of white matter in N3H mice up to 56 days after cerebral ischemia.

Our study demonstrates that chronic supplementation of n-3 PUFA-enriched fish oil significantly improves the cortical concentration of n-3 PUFAs and ratio of n-3/n-6. Long-term neurological and histological protection and survival was achieved by dietary n-3 PUFAs supplementation in aged mice. n-3 PUFAs promoted post-stroke neurogenesis and angiogenesis and also reduced the white matter injury, correlating with improved sensorimotor functions. n-3 PUFAs are potential neuroprotective reagent to be translated into clinical use for old individuals. The dietary administration of n-3 PUFAs may contribute to protect the aged brain against ischemic stroke, facilitate recovery of neurological functions and alleviate mortality after ischemia in aged human beings.

## 

Supplemental Figure 1 n-3 PUFAs diet supplementation bolsters neurogenesis following cerebral ischemia in aged mice **(A, B)** Shown are representative images of doublecortin (DCX) and BrdU double-label immunofluorescence in the striatum after MCAO. Fields were chosen in the blue boxes indicated in Fig. 3C. Three-dimensional confocal scan of BrdU^+^/DCX^+^ cells in the striatum at 56 days after MCAO. Scale bar=50 μm (A), and scale bar=10 μm (B). **(C)** Representative confocal images showing the close localization of DCX^+^ cells along CD31^+^ vessels in striatum at 56 days after MCAO. White arrow indicates the DCX^+^ cells associated with the vessels. Scale bar=15 μm. **(D)** Quantification of DCX^+^ cells and DCX^+^/BrdU^+^ double-labeled cells at 56 days post ischemia. Data are presented as mean ± SEM, n=5 per group, *p≤0.05 vs. N3L. **(E-F)** Correlation of the left turns in the corner test **(E)** and forelimb preference in the cylinder test **(F)** at 21-35 days after MCAO with the number of DCX^+^ cells in striatum at 56 days after MCAO. N3L group, n = 5; N3H group, n=6.
