# Supplementary figures and images for: Promoting Neurovascular Recovery in Aged Mice after Ischemic Stroke - Prophylactic Effect of Omega-3 Polyunsaturated Fatty Acids
Source: Aging Dis. 2017 Oct 1;8(5):531–45. doi: 10.14336/AD.2017.0520 (PMC5614319; doi:10.14336/AD.2017.0520)

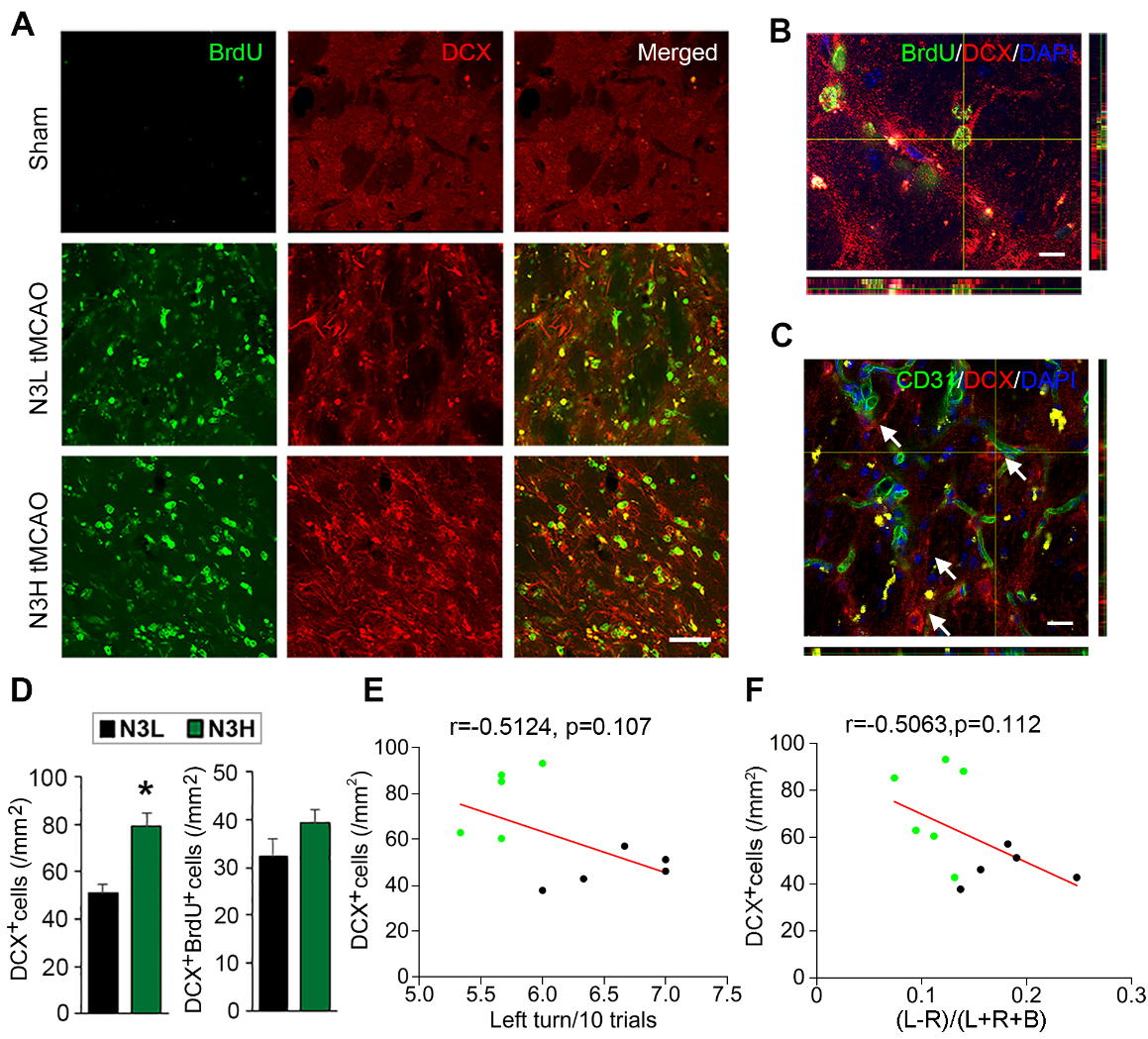

Supplement: Supplemental Figure 1 — (A, B) Shown are representative images of doublecortin (DCX) and BrdU double-label immunofluorescence in the striatum after MCAO. Fields were chosen in the blue boxes indicated in Fig. 3C. Three-dimensional confocal scan of BrdU+/DCX+ cells in the striatum at 56 days after MCAO. Scale bar=50 μm (A), and scale bar=10 μm (B). (C) Representative confocal images showing the close localization of DCX+ cells along CD31+ vessels in striatum at 56 days after MCAO. White arrow indicates the DCX+ cells associated with the vessels. Scale bar=15 μm. (D) Quantification of DCX+ cells and DCX+/BrdU+ double-labeled cells at 56 days post ischemia. Data are presented as mean ± SEM, n=5 per group, *p≤0.05 vs. N3L. (E-F) Correlation of the left turns in the corner test (E) and forelimb preference in the cylinder test (F) at 21-35 days after MCAO with the number of DCX+ cells in striatum at 56 days after MCAO. N3L group, n = 5; N3H group, n=6. [file ad-8-5-531-g6.tif]
